# Expression of a FRET-based ATP Biosensor in the *C. elegans* Intestine

**DOI:** 10.17912/micropub.biology.000284

**Published:** 2020-07-30

**Authors:** Jose Soto, Madeline Rivera, Gina Broitman-Maduro, Morris F Maduro

**Affiliations:** 1 Biochemistry Undergraduate Major; 2 MARCU-STAR Program; 3 University of California, Riverside; 4 Department of Molecular, Cell and Systems Biology

**Figure 1:**
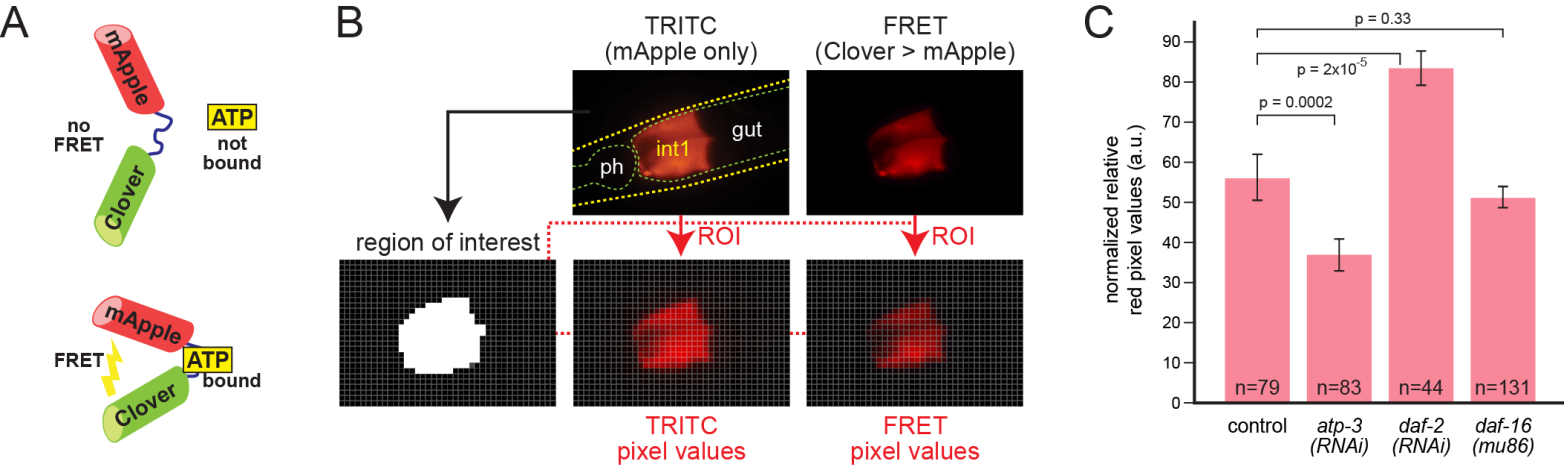
**A.** Principle of the FRET-based ATP sensor, after Zhang *et al.*, 2018. When not bound to ATP, the mApple and Clover fluorochromes are physically far apart. Clover is a form of green fluorescent protein that is excited by blue light and emits green light. mApple is a form of red fluorescent protein that is excited by green light and emits red light. The blue excitation light used for Clover produces only minimal excitation of mApple. However, when ATP is bound, the two fluorescent portions of the sensor are brought close together, permitting emission from Clover to directly excite mApple by FRET. Hence, the amount of red emission from the sensor protein, due to blue excitation, is an indirect measurement of the fraction of protein that is bound to ATP. As a control, excitation/emission of the Clover or mApple portions can detect the sensor protein independent of its interaction with ATP. In our hands, detection of mApple produced a much higher signal-to-noise ratio because of lower red autofluorescence in the gut. **B.** Image acquisition and analysis. The anterior gut was imaged twice with a 63x objective. The first image used direct excitation and emission of mApple using a TRITC filter set to assess where the sensor protein was present. The second image was obtained using a FRET filter set. This allows excitation of Clover, which produces green emission that excites mApple which is detected as red emission. Because the FRET emissions are of a much lower intensity, the image shown here was enhanced for contrast. Using a Python script, the TRITC image was used to identify 100×100 pixel blocks that contain the sensor protein, and hence define a region of interest (ROI). Pixel values were obtained in the red channel for the TRITC and FRET images across the ROI to generate an average red pixel value ratio. Abbreviations: ph, pharynx; int1, first intestinal ring. **C.** Histograms showing the data for control and test cases. Error bars denote SEM. (a.u. = arbitrary units)

## Description

The *C. elegans* intestine is a major site of the generation and storage of chemical energy. Adenosine triphosphate (ATP) is a major molecule involved in energy transfer, hence regulation of intracellular ATP levels is a critical aspect of metabolism. Recently, ATP sensor proteins have been used to indirectly measure ATP levels in living cells (Zhang *et al.*, 2018). These sensors are derived by inserting the ε subunit of the bacterial F_0_F_1_-ATP synthase between two fluorescent protein domains, allowing detection of protein bound to ATP by Förster Resonance Energy Transfer, or FRET (Imamura *et al.*, 2009). Others have recently demonstrated use of FRET-based ATP sensors in the *C. elegans* pharynx and muscle (Tsuyama *et al.*, 2013; Wang *et al.*, 2019). Here we test the intestinal expression of a similar FRET-based ATP sensor derived from the green/red fluorochrome pair Clover/mApple (Figure 1A) (Mendelsohn *et al.*, 2018). When the protein is bound to ATP, the two fluorochromes are brought into proximity, permitting emission from Clover to excite mApple (Imamura *et al.*, 2009; Mendelsohn *et al.*, 2018).

We made transgenic animals expressing the sensor under the control of the *pept-1* promoter, which in arrays drove strongest expression in the anterior intestinal ring, int1 (Mendelsohn *et al.*, 2018; Nehrke, 2003). We measured FRET by comparing red signal in image pairs as described in Figure 1B. Individual adult animals were imaged twice in the same focal plane. In the first image, mApple was detected by a TRITC filter set to reveal the sensor location independent of ATP levels. A second image was obtained using a filter set to excite Clover and detect red FRET emission from mApple. We devised an automated image analysis pipeline in Python to rapidly compute average ratios of FRET:mApple signal for each image pair and combine the data across multiple animals.

We tested three genetic conditions to evaluate whether the sensor produces similar relative ATP levels as previously determined by others (Fig. 1C). Knockdown of the nuclear-encoded ATP synthase subunit gene *atp-3* by RNAi results in a decrease of 60-80%, and knockdown of *daf-2*, an increase of 100-200%in biochemical ATP levels in whole animals, depending on their age (Dillin *et al.*, 2002). In contrast, mutation of *daf-16* produces little or no change in ATP levels (Braeckman *et al.*, 1999). Mutation of *daf-2* produced a modest increase in muscle ATP levels after several days, measured by a similar FRET-based ATP sensor, ATeam (Wang *et al.*, 2019). As shown in Fig. 1C, our results were qualitatively similar. However, the differences seen with the sensor, while statistically significant, are of smaller magnitude, i.e. only a 40% decrease in *atp-3(RNAi)* and a 50% increase in *daf-2(RNAi)*. Conservatively, therefore, an *in vivo* ATP biosensor is best used to compare relative free ATP levels across genotypes and conditions, and not to directly infer actual biochemical changes.

Our results suggest that even with a modest low-cost fluorescence setup, an *in vivo* ATP FRET reporter can be a useful way to measure aspects of metabolic state in the *C. elegans* intestine. Improvements in expression of the reporter and the use of confocal microscopy are likely to further increase the usefulness of this system.

## Methods

*Construction of the sensor transgene*. We obtained plasmids encoding the normal and kinase-dead versions of the novel Clover-ATP-mApple fusion protein (Mendelsohn *et al.*, 2018). These were used for PCR and Gibson assembly to fuse 1407 bp of the *pept-1* promoter to the sensor coding region and the 3’UTR of *unc-54* from vector pPD95.67.

Strains and worm handling. *unc-119(ed4)* mutants were made transgenic for the *pept-1*-driven sensor and an *unc-119(+)* rescue plasmid by microinjection. Arrays were integrated to generate MS2495 strain carrying *irIs158* (normal sensor) and MS2499 carrying *irIs162* (kinase-dead sensor). RNAi was performed by feeding of the HT115 bacteria carrying the empty vector, *daf-2* or *atp-3* sequences using standard methods (Kamath and Ahringer, 2003).

*Fluorescence microscopy.* The anterior intestines of 1- to 2-day old adults were imaged on an Olympus BX-51 upright epifluorescence microscope equipped with a 100W mercury arc lamp and Canon EOS 77D camera with LMScope C-mount adapter. The mApple was detected using a Chroma 31002 TRITC filter set. For FRET detection we combined the HQ500/20x exciter and Q515lp beam splitter from a Chroma 41029 YFP filter set with a 635/20 emission bandpass filter (a gift from Dr. David Carter, UCR Microscopy Core). To minimize variability in fluorescence intensity, images for a set of experiments were acquired within a short time using the same mercury bulb and settings.

*Image analysis*. Images of size 2656×3984 pixels were taken at ISO 100 and ¼ sec exposure and saved in JPG format using Canon Digital Photo Professional. In our first studies, we opened image pairs as layers in Adobe Photoshop and use the control TRITC image to identify a region of interest. We would then extract pixel values of this region from both images. We developed a Python script to process image pairs automatically (available at https://github.com/MaduroMF/FRETcalc/releases/tag/1.0). The script subdivides the first image into 100×100 pixel blocks, and if the average red pixel value of a block is higher than a background of 50, average red pixel values are recorded from the same block between the control and FRET images. For each image pair the program computes both the average of the ratios obtained across the regions of interest, as well as the ratio of the average pixel values. For the histogram shown in the figure the latter was used to compute the means, standard error, and *p* values using a two-sided *t* test. The pixel ratio (*r*) values were scaled for the histogram by computing (*r*-0.04)/0.04*100. The subtracted ratio of 0.04 was obtained from measurements of background FRET from a kinase-dead version of the sensor.
